# Structured Didactic Teaching Sessions Improve Medical Student Neurology Clerkship Test Scores: A Pilot Study

**DOI:** 10.2174/1874205X00802010008

**Published:** 2008-04-23

**Authors:** Daniel L Menkes, Mary Reed

**Affiliations:** Department of Neurology, University of Tennessee health Sciences Center at Memphis, 855 Monroe Avenue, Link Building, Room 415, Memphis, TN 38163, USA

**Keywords:** Medical student, education, didactic sessions, feedback, test performance

## Abstract

OBJECTIVE: To determine the effectiveness of didactic case-based instruction methodology to improve medical student comprehension of common neurological illnesses and neurological emergencies. SETTING: Neurology department, academic university. PARTICIPANTS: 415 third and fourth year medical students performing a required four week neurology clerkship. MAIN OUTCOME MEASURES: Raw test scores on a 1 hour, 50-item clinical vignette based examination and open-ended questions in a post-clerkship feedback session. RESULTS: There was a statistically significant improvement in overall test scores (p<0.001). CONCLUSION: Didactic teaching sessions have a significant positive impact on neurology student clerkship test score performance and perception of their educational experience. Confirmation of these results across multiple specialties in a multi-center trial is warranted.

## INTRODUCTION

Medical educators agree that teaching clinical reasoning skills is of paramount importance. However, there is no general consensus on the best methodologies for assessing whether or not these skills have been acquired (Epstein RM New Engl J Med 2007) [1]. Previous methods have included student feedback, standardized curricula and supervised instruction on a specific procedural skill (Ende J JAMA 1983) [2] (Wang TS Arch Dermatol 2004) [3]. A systematic literature review produced no study that demonstrated a quantifiable objective improvement in student test score performance after a specific teaching intervention. For this reason, we conducted this study in order to assess the effectiveness of biweekly didactic instruction sessions as measured on a written examination specifically designed to test clinical reasoning abilities.

## METHODS

The examination scores of all 415 medical students who successfully completed their required Neurology Clerkship were retrospectively analyzed for the one year before and the one year after the didactic teaching sessions were instituted. The clerkship was four weeks in length and consisted of rotations on an inpatient neurology service at one of the three affiliated teaching hospitals. Every student enrolled in the clerkship was required to attend an orientation session wherein the clerkship handout was reviewed such that all students were aware of the criteria by which they would be evaluated. There were never any more than 5 students assigned to a particular hospital service and never more than 15 students per clerkship in total. As an incentive to attend the didactic sessions, students who were present for every didactic session were allowed to take a five question bonus examination; all but two of the post-intervention group was eligible. Restated, 145 of 147 students attended all of the eight didactic sessions. However, the bonus examination scores were not assessed as the pre-intervention group was not given this option. All of the students took a 50-item test that required them to select the best answer from among four choices within one hour. Each student had to make a diagnosis from a clinical context and select the appropriate laboratory test, imaging study or medication that would address that diagnosis. The students were also informed that the test would be changed on a yearly basis and were bound to the university’s honor code so that communication regarding the test's contents would be minimized. Only two questions differed between the two years although all the students were blinded to this fact. Both groups of students were informed that approximately half of the examination would test neurological emergencies, (e.g. status epilepticus, acute stroke), whereas the remainder would evaluate common neurological diagnoses, (e.g. headache, neuromuscular disorders). With the exception of the first author, none of the instructors were aware of the actual test questions so that they could not “coach” the students on the correct answers.

The first group of students was not given any formal, standardized didactic instruction. In contrast, the subsequent year's students were required to attend formal didactic teaching sessions lasting 90 minutes, which were taught by both private practitioners and academicians, on a biweekly basis for four consecutive weeks. All vignettes were based on actual patients that the lead author had encountered in clinical practice. The list of topics is included in Table **1**.

All of the neurologists leading the discussion were given both the diagnosis and the specific teaching points to cover with each case. In contrast, the students received neither. An example of a case involving the Guillain-Barré syndrome with the teaching points appended, (the instructor’s version), is as follows:

A 23-year-old man is referred to your office with a three day history of Bell’s palsy. He states that he noticed that the right side of his face was drooping 3 days ago. He denies any headache, nausea, vomiting, vertigo or tinnitus. He has no past medical history except that he was evaluated in the emergency room 20 days earlier for diarrhea and dehydration. He received 2 liters of normal saline and was discharged with a prescription for Imodium. His diarrhea resolved 2 days later. On examination, he is afebrile with normal vital signs. His neurological examination is remarkable for an infranuclear VIIth nerve weakness. The Weber test does not lateralize and the Rinné test is normal bilaterally. Taste sensation is equal on both sides of the tongue. His motor examination demonstrates 4/5 weakness in hip flexors and extensors bilaterally. He is globally areflexic. His sensory examination demonstrates decreased vibration at the ankles. He has a slight sensory ataxia on tandem gait testing. [Diagnosis: Guillain-Barré syndrome].

- Discuss why this is not a case of Bell’s palsy.
- Discuss why this could not represent a NMJ disorder or a myopathy
- Discuss why this is an acquired rather than an inherited neuropathy
- Discuss why this is a demyelinating rather than an axonal neuropathy
- Discuss the importance of his previous diarrheal illness
- Discuss the most likely diagnosis and how this could be assessed
- Discuss the possible treatment options with respect to risk and benefit

The students were asked to localize the lesion in the nervous system, discuss the pathophysiology, and understand basic diagnostic testing and treatment concepts. The neurologist leading the discussion was directed to discuss his or her thought process in localizing the lesion so that the students could refine their own localization skills. The instructor was then directed to pose questions to the students as outlined in the discussion points so that the students could learn the nuances of neurological reasoning. All the discussion leaders were advised that the students needed to answer the questions initially in order to determine where the students were having difficulties so that these finer points could be clarified. In addition to these didactic sessions, the post-intervention group students were also given access to a PowerPoint presentation that reviewed these principles as well as a list of neurological emergencies and their evaluation.

The data were collected by one of the authors (MR), who was blinded to the hypothesis. A two-tailed Student’s t-test for unequal group size and variance was used to assess for statistically significant differences in the mean scores between these two groups at a 95% confidence interval. The same analysis was applied to assess for gender differences as well as for differences between third and fourth-year students.

The students were also administered open-ended questionnaires before and after the intervention that asked open ended questions regarding “test fairness,” “quality of the clerkship experience” and “suggestions for improvement.” These sessions were attended only by MR so that the students would speak freely in the absence of the clerkship director (DM). These non-quantitative data were analyzed retrospectively in order to ascertain the students' perception of the didactic teaching sessions and the quality of their educational experience. Although quantitative data are not available, the students in the pre-intervention group generally gave an unfavorable impression of the clerkship. The salient features of their comments were that their educational experience was skewed toward inpatient neurological diseases and that their educational experiences were uneven as they depended on the interests of the attending physician. Post-intervention, the overall impression was favorable in that the students stated that their educational experience was more uniform and they were exposed to the management of outpatient neurological disorders. Of note, the clerkship director received no teaching awards for the clerkship before the intervention and a teaching award for the year after the intervention. Student feedback continues to be positive since the initiation of these didactic teaching sessions. The clerkship director (DM) has continued to receive a teaching award every year since these changes were implemented.

## RESULTS

This study’s data are summarized in Table **2** as follows:

The only statistically significant differences between the two groups were that the interventional group had both fewer students and a smaller proportion of fourth year medical students. Only two students in the intervention group were not present at all the didactic teaching sessions for an overall compliance rate of 99%. The average percentage of correct responses was 74% before the intervention and 76% after the intervention (p<0.001). Sub-group analysis revealed that this difference was attributable to an improvement in the third-year medical students' scores. Although there was a trend towards improvement in the fourth year students' scores, this did not reach statistical significance. While women scored marginally better on the standardized test, this did not reach statistical significance either.

A review of the student comments before and after the intervention indicated that the students expressed a greater degree of satisfaction with respect to “fairness of the examination,” “meeting clerkship goals” and “acquiring the skills required for clinical practice.” An analysis of student feedback revealed that the students had a more favorable impression of the Neurology Clerkship after the didactic sessions were implemented. The students were in general agreement that the teaching sessions were of greater value than any printed material.

## DISCUSSION

This study demonstrates the positive effects of didactic teaching sessions on medical student test performance in a Neurology Clerkship. The students also expressed a greater degree of satisfaction with their clerkship experience as well. Several issues merit discussion.

Sub-group analysis demonstrated that the significant improvement was primarily attributable to the third-year students. This is particularly notable because there were significantly fewer fourth-year students in the post-intervention group. It is generally assumed that fourth year students would test better than third year students given one more year of clerkship and clinical experience. If this were true, the teaching intervention would have to be especially effective if the average test score improved in the post-intervention group given the higher proportion of third year students in the latter. Although the third-year students performed less well than their fourth-year counterparts before the intervention, no statistically significant difference between third and fourth year students could be detected after the intervention. Moreover, the average score of the third year students was marginally better than the fourth-year student scores after the educational intervention. For these reasons, the data indicate that student test performance can be improved with didactic teaching sessions such that a third year medical student will perform as well as a fourth year student in a neurology clerkship.

Although statistically significant, the increase in aggregate test scores was not marked; 74% versus 76%. Despite the students being given various types of study materials, (e.g. study guidelines, PowerPoint summaries and didactic sessions), they still continued to select a wrong answer 25% of the time. This may be a cause for concern as the students were given an examination specifically designed to test neurological emergencies and commonly encountered neurological diagnoses. These results suggest that ongoing continuing medical education in neurology may be necessary in order to insure that these concepts are retained during post-graduate medical practice. A previous report noted that orthopedic surgery residents who took additional subspecialty elective training as a medical student continued to outperform their peers both as first year and as chief residents (Freeman KB Journal of Bone and Joint Surgery 1998) [4]. For this reason; we recommend that other clerkships make a concerted effort to re-emphasize basic concepts learned in other core clerkships in addition to their own.

We recommend exposing students to a required neurology clerkship in the third year for a variety of reasons. First, our study demonstrated that the didactic teaching sessions compensated for the difference in clinical experience between the third and fourth year students. Earlier exposure to this specialty might also increase the number of medical students who choose neurology as a specialty. One article projected that our nation will have only 60% of the neurologists needed by the year 2010 (Kurtzke JF Neurology 1986) [5]. Therefore, many patients will rely on non-neurologists to recognize and treat common neurological illnesses and emergencies. For this reason, any method that improves a student's knowledge and clinical skills in this discipline should be initiated. Exposure to neurology earlier in the medical school curriculum would provide additional opportunities for neurology instruction in related specialties during the students' fourth year even for students who do not choose to practice neurology.

The medical students' perception of the quality of their education is also important although it is less quantifiable. Student feedback regarding these didactic teaching sessions was almost uniformly positive. Specifically, the students were of the opinion that they were exposed to a wider range of subject material. However, a review of their direct patient exposure revealed a strong bias towards inpatient disorders such as stroke and seizure disorders. Because neurology is primarily an outpatient and consultative specialty, students who do not received targeted, didactic instruction may not develop a clinical approach to common neurological disorders such as vertigo, headache, and neuromuscular diseases, which are generally treated on an outpatient basis. However, we are of the opinion that these didactic sessions should address actual case histories so that the students are given examples of patients that will be encountered in clinical practice. These sessions allow a clinician to explain the clinical reasoning process as well as the salient features of the disease process.

There are several limitations of this study that merit discussion as well. This was a study that was purposefully conducted retrospectively. Despite the limitations of a retrospective study, a prospective study of student test score performance would have not necessarily been superior and may have raised some ethical issues. When students know that they are being observed for test performance, the confounding variable of a “Hawthorne effect” cannot be excluded. Furthermore, medical education presumes that all students should be given equal access to educational opportunities. It may be unethical to conduct a trial in which students were randomized to receive didactic instruction or no instruction at all. This may be especially true for students applying to competitive specialties that require maximizing one’s grade point average. Another limitation regards the accuracy of historical controls. The pre-intervention group differed in that fourth year students were more highly represented. However, these students tend to have more medical knowledge, clinical reasoning skills and better test-taking skills than their third year counterparts. Nonetheless, the superior performance of the post-intervention group, comprised mostly of third year medical students, make this study’s findings even more remarkable. This study did not address alternative educational methods such as “e-learning” or simulated patients. While these methods may be beneficial as well, the student feedback clearly indicated that they perceived a superior educational experience when being instructed by a clinical neurologist.

In conclusion, a twice-weekly didactic teaching session based upon actual patient case histories improved final neurology clerkship examination scores and overall student satisfaction. A multi-institutional study that would confirm the results of this pilot study is warranted. Similar studies conducted in other medical specialties are also indicated in order to validate this study.

## Figures and Tables

**Table 1.** 

Week	Topics
1	Cerebrovascular disease Encephalopathies
2	Headaches and Brain tumors Dizziness, Vertigo, Hearing Loss and Syncope
3	Movement disorders Myelopathies and Central Demyelinating Disease
4	Seizure and Sleep Disorders Neuromuscular diseases

**Table 2.** 

	Before	After	p value
n	268	147	NS
n compliant	N/A	145	NS
Males	162 (60%)	88 (60%)	NS
4^th^year students	156 (58%)	31 (21%)	<0.05
All test scores (mean ±S.D.)	36.9 ±3.7	38.3 ±3.6	<0.001
4^th^year scores (mean ±S.D.)	37.1 ±3.4	38.0 ±3.8	NS
